# P-1705. Infectious Diseases Pharmacist Curbsides: Questions Infectious Diseases Providers Ask Infectious Diseases Pharmacists

**DOI:** 10.1093/ofid/ofae631.1871

**Published:** 2025-01-29

**Authors:** Matthew Davis, Julia Kufel, Wesley D Kufel, Jennifer Ross, Louise-Marie Oleksiuk, Rachael Ours, Ethan A Smith, Christine Pham, Michael J Trisler, Frank Tverdek

**Affiliations:** Infectious Diseases Connect ; UPMC, Austin, Texas; St. Joseph's Health, Syracuse, New York; Binghamton University School of Pharmacy Sciences, Binghamton, NY; M Health Fairview - University of Minnesota Medical Center, Minneapolis, Minnesota; University of Pittsburgh Medical Center, Pittsburgh, PA; UPMC, Erie, Pennsylvania; Ronald Reagan UCLA Medical Center, Los Angeles, California; University of California, Los Angeles; David School of Medicine/University of California, Los Angeles, Los Angeles, California; UPMC Presbyterian Shadyside, Pittsburgh, Pennsylvania; Fred Hutchinson Cancer Center, Seattle, Washington

## Abstract

**Background:**

Infectious Diseases (ID) consult services, typically led by ID physicians, benefit significantly from the support of ID pharmacists. These pharmacists, with specialized training in ID pharmacotherapy through post-graduate residencies or fellowships, play a crucial role in advising on the selection, dosing, duration, and monitoring of antimicrobials. While the contribution of ID pharmacists to antimicrobial stewardship programs is well described, their impact in supporting ID consult services remains underdocumented. This report explores the clinical inquiries made by ID physicians and advanced practice providers (APP) to ID pharmacists on consult services, detailing how these interactions influence patient treatment and monitoring plans.

**Figure 1:**
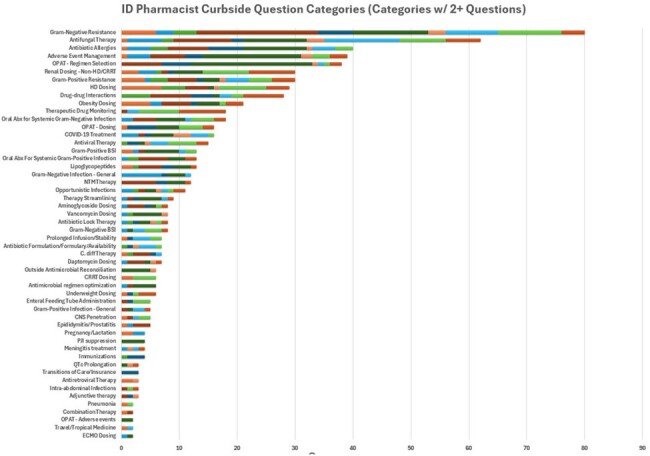
ID Pharmacist Curbside Question Categories (Categories with 2+ Submissions)

**Methods:**

Ten ID residency- or fellowship-trained clinical pharmacists practicing in various practice settings documented questions they received from ID providers (attendings, fellows, and APPs) for patients on their respective ID consult service over a 6-month period. The impact of the conversations was documented if the pharmacist recommending a new drug, dose, duration, or monitoring plan which resulted in a change in care. Additional information was captured on the time spent answering questions, category of question, and pertinent microbiology or organism phenotype to describe areas of highest need for ID pharmacist support.

**Figure 2:**
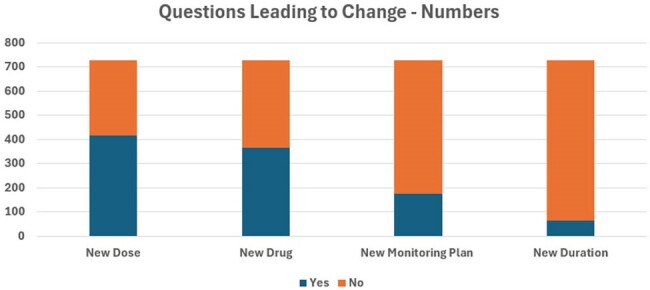
Questions Leading to Therapeutic Change

**Results:**

Over the first three months of documentation, 782 questions were asked by ID providers to ID pharmacists. Total direct time spent answering questions was 8,937 minutes. The top three question categories were gram-negative resistance, antifungal therapy, and antimicrobial allergies. Seven hundred and three (89.8%) questions resulted in a clinical change in management as recommended by the ID pharmacist. The most common clinical change category was recommending a new dose which occurred in 57.4% of questions followed by new antimicrobial 50.4%, new monitoring plan 24.2% and new duration of therapy 8.8%.

**Conclusion:**

ID pharmacists offer critical support that frequently changes management on ID consult services. Integrating their expertise into a multidisciplinary ID team is essential for achieving optimal patient safety and treatment outcomes.

**Disclosures:**

**Wesley D. Kufel, Pharm.D., BCPS, BCIDP**, Merck & Co.: Grant/Research Support|Shionogi, Inc: Grant/Research Support **Jennifer Ross, PharmD, BCIDP**, Shionogi Inc.: Advisor/Consultant **Frank Tverdek, PharmD**, Merck: Advisor/Consultant

